# Relationship of pulmonary toxicity and carcinogenicity of fine and ultrafine granular dusts in a rat bioassay

**DOI:** 10.3109/08958378.2011.594458

**Published:** 2011-08-05

**Authors:** Angelika Kolling, Heinrich Ernst, Susanne Rittinghausen, Uwe Heinrich

**Affiliations:** Fraunhofer Institute for Toxicology and Experimental Medicine (ITEM), Hannover, Germany

**Keywords:** Rats, histopathology, pulmonary neoplasms, inflammation, granular dusts

## Abstract

The current carcinogenicity study with female rats focused on the toxicity and carcinogenicity of intratracheally instilled fine and ultrafine granular dusts. The positive control, crystalline silica, elicited the greatest magnitude and progression of pulmonary inflammatory reactions, fibrosis and the highest incidence of primary lung tumors (39.6%). Addition of poly-2-vinylpyridine-N-oxide decreased inflammatory responses, fibrosis, and the incidence of pulmonary tumors induced by crystalline quartz to 21.4%. After repeated instillation of soluble, ultrafine amorphous silica (15 mg) a statistically significant tumor response (9.4%) was observed, although, the inflammatory response in the lung was not as persistently severe as in rats treated with carbon black. Instillation of ultrafine carbon black (5 mg) caused a lung tumor incidence of 15%. In contrast to a preceding study using a dose of 66 mg coal dust, lung tumors were not detected after exposure to the same coal dust at a dose of 10 mg in this study. Pulmonary inflammatory responses to coal dust were very low indicating a mechanistic threshold for the development of lung tumors connected with particle related chronic inflammation. The animals treated with ultrafine carbon black and ultrafine amorphous silica showed significantly more severe lesions in non-cancerous endpoints when compared to animals treated with fine coal dust. Furthermore, carbon black treated rats showed more severe non-cancerous lung lesions than amorphous silica treated rats. Our data show a relationship between tumor frequencies and increasing scores when using a qualitative scoring system for specific non-cancerous endpoints such as inflammation, fibrosis, epithelial hyperplasia, and squamous metaplasia.

## Introduction

Over the past 25 years an increasing number of chronic inhalation and intratracheal instillation studies have been conducted in rats to assess the toxicity of various dust particles including ultrafine carbon black (Nikula et al., 1995; Heinrich et al., 1995; Mohr et al., 2006), fine coal dust (Martin et al., 1977; Lewis et al., 1989; Pott et al., 2000; Pott et al., 2003; Mohr et al., 2006), fine crystalline SiO_2_ (Pott et al., 2000; Pott et al., 2003) and ultrafine amorphous SiO_2_ (Pott et al., 2003; Mohr et al., 2006). These studies demonstrated that chronic inhalation or repeated instillation of particles result in chronic inflammation, fibrosis, epithelial hyperplasia and with poorly soluble particles (PSP) late in life also in primary pulmonary neoplasias.

The question of carcinogenicity induced by various dusts at high concentrations has already been discussed in various papers (Mauderly, 1997; Mossman, 2000; Nikula, 2000; Donaldson and Lang Tran, 2002; Driscoll et al., 2002; Schins et al., 2002; Schins et al., 2007). Several dust particles have been shown to elicit tumors in experimental animals. These include poorly soluble dusts with low toxicity such as carb on black as well as dusts with high toxicity such as crystalline silica particles (for reviews see references [Greim et al., 2001; Borm et al., 2004; Knaapen et al., 2004]). Crystalline silica, inhaled from occupational environments, has been classified as a human carcinogen by the International Agency for Research of Cancer (IARC, 1997). According to IARC the overall data from cancer studies in rodents exposed to carbon black provided sufficient evidence of carcinogenicity. It was evaluated as possibly carcinogenic to humans, Group 2B (Baan, 2007).

Ultrafine amorphous silica a particle with a certain solubility have until now not been shown to initiate the development of lung tumors in the rat lung (Pott et al., 2003; Mohr et al., 2006). After 13 weeks inhalation exposure to crystalline and amorphous silica using exposure concentrations that resulted in similar inflammatory responses in the rat lung for both types of silica only crystalline silica exposed rats showed an increased mutation frequency in lung epithelial cells (Johnston et al., 2000). IARC determined that amorphous silica particles were not classifiable as to its carcinogenicity to humans (category III) but it was noted that the evaluation based on the lack of toxicological and epidemiological data for this material (Warheit, 2001; Merget et al., 2002).

Another issue is the interpretation of findings considering the carcinogenic hazard of coal dust. Previous inhalation studies (Nikula et al., 1997; Busch et al., 1981; Lewis et al., 1989) in rats exposed to coal dust (Nikula et al., 1997: 2mg/m^3^, 7h/day, 5 days/week, 24 months; Busch et al., 1981: at 6.6 and 14.9 mg/m^3^ 6h/day, 5 days/ week for up to 20 month) did not show an excess of lung tumors.

Lung tumors have been found after inhalation of high concentrations (200 mg/m^3^) of coal dust (Martin et al., 1977) in rats although the incidence was not statistically significant due to a limited number of control rats.

Also,lung tumors have been found after repeated intratracheal instillation of coal dust (6 mg coal dust once a week for 11 weeks) in rats with an incidence of 57.4%. (Pott et al., 2000).

So far, coal mine dust is not considered as a human carcinogen (IARC category III). Human exposure to coal dust is associated with increased risk of nonmalignant effects in the lung, such as restrictive and obstructive lung disease, but there is no conclusive evidence for increased lung cancer risk (Workshop Consensus Report; ILSI Risk Science Institute, 2000).

The objectives of this life time carcinogenicity study were to provide some information on particle induced lung lesions preceding particle induced lung tumor response as well as some guidance for hazard assessment on the interpretation of neoplastic and nonneoplastic responses of the rat lung to granular dusts with various toxicities, sizes and solubilities.

Based on the available data on the lung tumor inducing effect of fine coal dust with low content of quartz (< 1%) in rats only high particle doses in the lung seem to be able to induce lung tumors. Based on the results of a preceding 4 weeks- and 3-months study with coal dust (Ernst et al., 2002) investigating the degree of permanent lung inflammation at various particle doses and taking into account the coal dust doses employed by Pott et al. 2000 resulting in 57.4% lung tumor incidence, a more than six time lower total dose of coal dust than used by Pott et al. 2000 was applied by repeated intratracheal instillation in this study. The purpose of this experimental group was to see whether a coal dust induced low to moderate degree of permanent lung inflammation will still lead to the development of lung tumors.

Amorphous SiO_2_ (Aerosil 150®) was chosen as an ultrafine non-biopersistent dust dissolving in the lung with a rapid elimination rate (Ernst et al., 2002, Ernst et al., 2005) but, which is also known to be toxic and to produce transient lung damage (Murphy et al., 1998). The question was whether repeated application into the rat lung for a certain time of the lifetime (30 weeks) would be sufficient to induce lung tumors also with this type of toxic but soluble nanoparticle. For comparison, a biopersistent and non-soluble nanoparticle was chosen (carbon black (PRINTEX® 90).

Crystalline silica (Quartz DQ 12) beside being used as a positive control for particle induced lung tumors was also applied in the current study together with poly-2-vi-nylpyridine-N-oxide (PVNO) in one experimental group to investigate whether PVNO is able to reduce inflammatory and fibrotic lung responses (Ernst et al., 2005) and how this effect would change the tumor outcome.

## Materials and methods

### Test materials

#### Quartz

Dörentrup “ground product No. 12” (DQ 12), particle size 10% < 0.6 μm, 50% < 1.1 μm, 90% < 2.3 μm; BET surface area 9.4 m^2^/g; density 2.6 g/ml. Dispersion liquid: physiological saline (0.9% in H_2_O).

#### Amorphous SiO_2_

Aerosil® 150. Fluffy white powder, hydrophilic fumed silica CAS # 112945-52-5 ex. 7631-86-9 EINECS#231-545-4 Degussa (1984), average arithmetical diameter of primary particles 0.014 μm; BET surface area 150 ± 15 m^2^/g; density ∼2.2 g/m^2^; 99.8% SiO_2_. Dispersion liquid: physiological saline (0.9% in H_2_O).

#### Carbon black

PRINTEX 90® (Degussa, Germany), average arithmetical diameter of primary particles 0.014 μm; BET surface area ∼300 m^2^/g; density 1.8–1.9 g/m^2^ (Degussa, 1994). Dispersion liquid: physiological saline (0.9% in H_2_O), Tween 80®

#### Coal dust

Milled lean coal with crystalline SiO_2_ < 0.1%, ash 5%, density 1.4 mg/ml, particle size 50% < 4 μm (measured with Coulter Counter), BET surface area 4.1 mg^2^/g (Eickhoff, 2001). Dispersion liquid: physiological saline (0.9% in H_2_O), Tween 80®.

Poly-2-vinylpyridine -N-oxide (PVNO): Silicosis inhibitor, laboratory name “P 204” produced 1986 in the laboratory of Dr. Brockhaus, Medical Institute of Environmental Hygiene, Düsseldorf, Germany; 2% solution in saline.

Tween 80® (polyoxyethylene sorbitan monooleate) (Sigma-Aldrich, Germany, P-1754) was used as detergent (0.5%) for suspensions of carbon black and coal dust in saline.

### Animals

Female Wistar WU rats (Crl:WI(WU)BR), 6 weeks of age, were purchased from Charles River Deutschland, Sulzfeld, Germany. They were randomly assigned to the treatment groups and were approximately 8 weeks of age at the start of the first intratracheal instillation (for the purpose of testing carcinogenicity we used 50 rats per group. We added 9 more rats, in case further special examinations were required, leading to a total of 59 used animals overall). The animals were kept under conventional laboratory conditions: two rats per polycarbonate cage (800 cm^2^) with tap water and pelleted food (1324N spec. prepared, Altromin International, Lage, Germany) *ad libitum*, softwood bedding, room temperature 22 ± 2°C, relative humidity 55 ±15%, and 12 h dark/light cycling. Individual body weights were recorded once per week during the first 3 months, thereafter every 4 weeks up to the end of the study.

All procedures employed for animal care and handling have been performed in accordance to German animal welfare rights.

### Treatment

Rats were anesthetized by CO_2_:O_2_ (65 %:35%) and treated by intratracheal instillation of 0.3 ml particle suspensions in saline. To improve homogenicity, suspensions were ultrasonicated for 5 min; the suspensions were then kept homogeneous by permanent stirring during the administration period; rats were anesthesized for the intratracheal instillation procedure using CO_2_:O_2_ = 65%:35%. Doses of dusts and PVNO, intervals between applications for the lifetime carcinogenicity study (29 months) and number of rats per treatment group are described in Table 1. Additional details of the experimental design have been published by Ernst et al. (2005).

**Table 1:** Treatment groups of the carcinogenicity study

Treatment (intratracheal instillation, PVNO: subcutaneous injection)	No. of instillations x dose (at intervals of days or months, respectively)	Animals/group
Control, 0.9% NaCl-solution	10×0.3 ml (7 days)	50 (+ 9)**
Crystalline SiO_2_ (Quartz DQ12)	1×3 mg	50 (+ 9)
Crystalline SiO_2_ (Quartz DQ12) + PVNO	1 × 3 mg Quartz 7 ×20 mg PVNO s.c. (4 months)	50 (+ 9)
Amorphous SiO_2_ (Aerosil® 150, 0.014 μm*)	30 × 0.5 mg (14 days)	50 (+ 9)
Carbon black (PRINTEX®, 0.014 μm*)	10 × 0.5 mg (7 days)	50 (+ 9)
Coal dust (4 μm*)	10×1mg (7days)	50 (+ 9)

* Average arithmetical diameter of primary particles.

** Reserve animals for further possible special investigations in brackets.

### Histopathology

Intercurrently dying rats were necropsied immediately upon discovery; moribund animals and rats surviving until terminal necropsy at 29 months after first instillation were euthanized by an overdose of CO_2_ and subsequent exsanguination. The lungs were fixed by intratracheal infusion of a 10% neutral buffered formalin solution under 20 cm of water pressure. Following infusion fixation, the trachea was tied off and the lungs were stored in formalin for 24 h. They were trimmed according to Bahnemann et al. (1995), dehydrated, and 7 lung tissue specimens per rat (the dorsal and ventral halves of lobes 3 (caudal right lobe)) and 5 (left lobe [section no. 1–4]), lobe 1 (cranial right lobe [section no. 5]), lobe 2 (middle right lobe) and lobe 4 (accessory right lobe [section no. 6]) were embedded in 6 paraffin wax blocks (lobe 2 and lobe 4 embedded in the same block).

### Routine histological examination

Seven lung tissue specimens (6 sections) were sectioned at 3 μm in 328 rats (those who had survived for at least 52 weeks:55 rats of the control group, 53 rats of the group treated with crystalline SiO_2_ (quartz DQ12), 56 rats of the group treated with crystalline SiO_2_ (quartz DQ12) and PVNO, 54 rats of the group treated with amorphous SiO_2_ (Aerosil 150®), 59 rats of the group treated with carbon black (PRINTEX® 90) and 51 rats of the group treated with coal dust). Single sections of each specimen were stained with hematoxylin and eosin and evaluated by light microscopy. Additionally, histological sections of macroscopically visible nodules were prepared. If primary tumors of other tissues than the lung had metastasized to the lung, their organ of origin was examined as well.

Histological evaluation of multiple step sections at intervals of 250 μm (at least 60 H&E-stained sections of 3 μm per rat) through the entire lung of 112 female rats which had survived at least for >101 weeks was performed in addition to enhance the amount of information obtained on induced pulmonary effects. This interval enables the detection of tumors even with a diameter of only 0.25 mm. Tumors of a diameter less than 1mm probably are missed by routine evaluation. Details of these examinations have been published by Kolling et al. (2008).

Tumor diagnosis and classification followed the nomenclature for classification of lung tumors and pre-neoplastic lesions in the rat proposed by WHO/IARC. The diagnostic criteria were published by WHO/IARC (Dungworth et al., 1992a). Classification of squamous cell lesions was conducted in consideration of the terminology and criteria developed in a workshop sponsored by the “Deutsche Forschungsgemeinschaft” (Boorman et al., 1996). The applied diagnostic criteria conform to the criteria of INHAND (International Harmonization of Nomenclature and Diagnostic Criteria) recently published by Renne et al., 2009. The findings were recorded and tabulated using an on-line computer program (P.L.A.C.E.S., version 2000.1, Instem Life Science Systems, UK).

The degree of the alterations was categorized in 5 grades (0 = absence; 1 = very slight; 2 = slight; 3 = moderate; 4 = severe; 5 = very severe). Histograms of severity scores across groups and histological findings were performed.

### Statistics

Statistical evaluation of the histopathological findings was done by the P.L.A.C.E.S. system using two-tailed Fisher's test.

Kaplan-Meier survival analysis was used to compare survival times between the 6 experimental groups. The log rank statistics was applied to test for differences between the groups.

The Mann-Whitney-*U*-Test was performed as non-parametric test for assessing significant differences of the severity code of histological findings between two groups (control, quartz and coal dust against the remaining experimental groups). Statistical significance was defined as *p* < 0.05.

## Results

### Mortality

The calculation of cumulative mortality showed an age-specific increase of the mortality rate for all experimental groups after the 77^th^ week (Ernst et al., 2002). At the end of the 125^th^ week of the carcinogenicity study the calculated mortality ratio based on survival rates in weeks showed no significant differences among all experimental groups.

### Neoplastic lesions

The incidences of primary pulmonary tumors and pre-neoplastic lesions per treatment group after routine histological examination are summarized in Table 2. Exaggerated bronchiolo-alveolar hyperplasia and squamous cell metaplasia (both lesions graded severe and very severe with signs of cytological atypia) were considered as preneoplastic alterations according to Dungworth et al., 1992b (Table 2).

**Table 2:** Incidences of tumors, tumor types and preneoplasias in the lungs of female Wistar rats after intratracheal instillation of granular dusts

	Control	Crystalline silica Quartz DQ12	Crystalline silica Quartz DQ12 + PVNO	Amorphous silica Aerosil® 150	Carbon black PRINTEX® 90	Coal dust
No. of rats examined	55	53	56	53	59	51
No. of rats with lung tumors	0 (0%)^a^	21 (39.6%)	12 (21.4%)	5 (9.4%)	9 (15%)	0 (0%)
No. of rats with multiple tumors	0 (0%)	11 (21%)	4 (7%)	0 (0%)	2 (3.3%)	0 (0%)
Total number of lung tumors/group	0	46	18	5	13	0
Frequency of lung tumor types						
No. of rats with bronchiolo-alveolar adenoma	0 (0%)	6 (11.3%)	2 (3.6%)	2 (3.8%)	2 (3.4%)	0 (0%)
No. of rats with bronchiolo-alveolar carcinoma	0 (0%)	14 (26.4%)	6 (10.7%)	2 (3.8%)	6 (10.2%)	0 (0%)
No. of rats with cystic keratinizing epithelioma	0 (0%)	5 (9.4%)	2 (3.6%)	0 (0%)	1 (1.7%)	0 (0%)
No. of rats with squamous cell carcinoma	0 (0%)	7 (13.2%)	2 (3.6%)	1 (1.9%)	2 (3.4%)	0 (0%)
No. of rats with adenosquamous carcinoma	0 (0%)	0 (0%)	1 (1.8%)	0 (0%)	0 (0%)	0 (0%)
Frequency of preneoplasias graded severe/very severe with signs of cytological atypia						
No. of rats with bronchiolo-alveolar hyperplasia	1 (2%)^a^	23 (43.4%)	7 (21.4%)	1 (1.9%)	9 (15%)	1 (2%)
No. of rats with squamous cell metaplasia	0 (0%)	4 (7.6%)	5 (8.9%)	0 (0%)	4 (6.8%)	0 (0%)
Total number of rats with preneoplastic lesions	1 (2%)	23 (43.4%)	10 (17.9%)	1 (1.9%)	12 (20.3%)	1 (2%)

a Number of tumor-bearing rats expressed as a percentage of the total number of rats examined in this treatment group

The number of rats with lung tumors and preneoplastic lesions (hyperplasia/metaplasia with atypia) and the total number of lung tumors per group are presented. The prevalence of lung tumor types including bronchiolo-alveolar adenoma, bronchiolo-alveolar carcinoma, cystic keratinizing epithelioma, squamous cell carcinoma and adenosquamous carcinoma is shown in addition.

**Figure 1:**
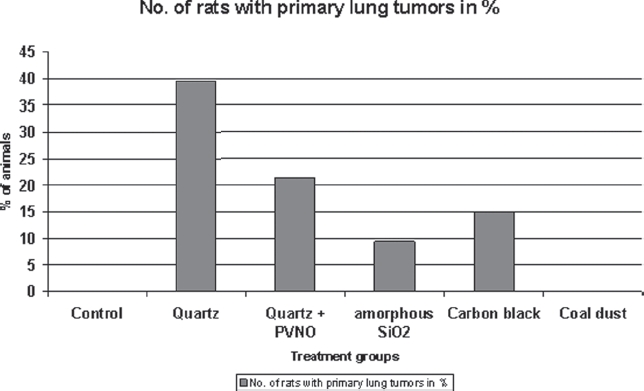
Incidences of primary lung tumors in the lungs of female Wistar rats after intratracheal instillation of granular dusts.

Exposure to quartz DQ12 caused the highest incidence of primary pulmonary tumors (21/53 [39.6%]), listed in decreasing incidence quartz DQ12 + PVNO (12/56 [21.4%]), Printex® 90—carbon black (9/59 [15%]) and amorphous SiO_2_—Aerosil® (5/54 [9.3%]). In the control group and in the group treated with coal dust no lung tumors were detected (Figure 1). Only one spontaneous preneoplastic lesion in a single control and coal dust treated animal each were detected as shown in Table 2.

The addition of step sections taken from the entire lung of 112 rats enhanced the tumor detection rate from 17 to a total number of 44 lung tumors in the quartz-treated rats, from 6 to 10 in the quartz + PVNO-treated rats, from 4 to 11 in the amorphous SiO_2_-treated rats, and from 4 to 10 in the carbon black-treated rats. Likewise no tumors were found in the lungs of the control group and of the coal dust-treated rats, which were examined by multiple step sections. These additional data confirmed the initial findings in all treatment groups. The results and additional details of the supplemental evaluation of step sections of this carcinogenicity experiment have been published elsewhere (Kolling et al., 2008).

### Non-neoplastic lesions

**Figure 2:**
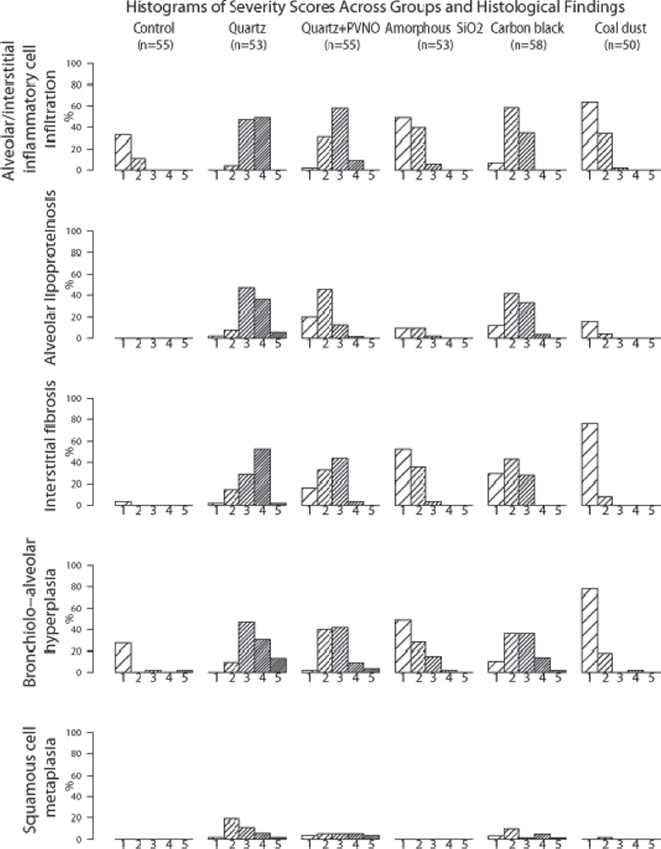
Histograms of severity scores across groups and histological findings Percent (%) of animals with the corresponding severity score of the histological finding 1: very slight 2: slight 3: moderate 4: severe 5: very severe.

**Figure 3:**
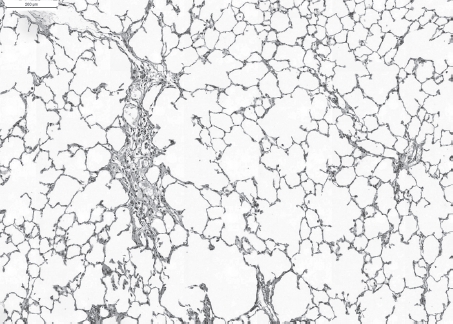
Slight (multi)focal interstitial fibrosis and inflammatory cell infiltration after repeated intratracheal instillation of ultrafine amorphous SiO_2_ (Aerosil®150). H&E × 50. (See colour version of this figure online at www.informahealthcare.com/iht)

**Figure 4:**
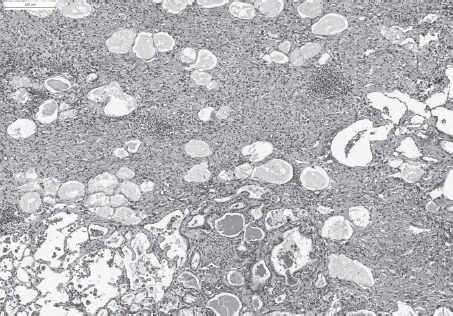
Severe fibrosis, inflammation and bronchiolo-alveolar hyperplasia after a single intratracheal instillation of Quartz DQ12. H&E, × 50. (See colour version of this figure online at www.informahealthcare.com/iht)

**Figure 5:**
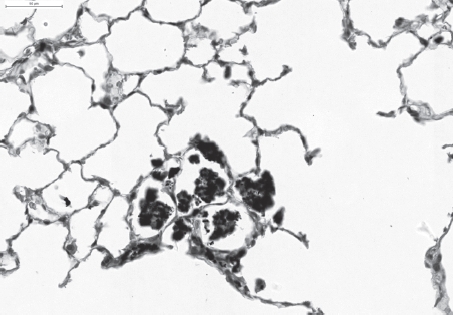
Slight multifocal intra-alveolar and interstitial accumulation of particle-laden macrophages associated with only minimal interstitial inflammatory cell infiltration after repeated intratracheal instillation of fine coal dust. H&E × 200. (See colour version of this figure online at www.informahealthcare.com/iht)

**Figure 6:**
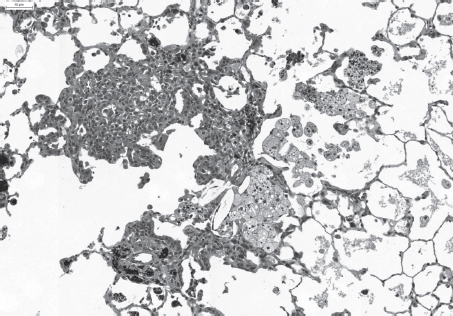
Slight multifocal accumulation of partly degenerating particle-laden macrophages, slight interstitial fibrosis, inflammatory cell infiltration and bronchiolo-alveolar hyperplasia after repeated intratracheal instillation of ultrafine carbon black (PRINTEX® 90). H&E × 100. (See colour version of this figure online at www.informahealthcare.com/iht)

Chronic exposure of female rats to the selected granular dusts caused particle-induced alterations in the lungs which were characterized by multifocal alveolar and interstitial accumulations of particle-laden macrophages, a mixture of bronchiolo-alveolar hyperplasia, multifocal mixed inflammatory cell infiltrations, interstitial fibrosis, alveolar lipoproteinosis, goblet cell and squamous cell metaplasia. Differences in severity and incidence were seen depending on treatment regimen and compound tested (Figures 2–6).

Essential features were intra-alveolar and interstitial inflammation accompanied by various degrees of bronchiolo-alveolar epithelial hyperplasia. A main component was the multifocal alveolar and interstitial accumulation of particle-laden macrophages with signs of degeneration and necrosis and various degrees of rupture seen in the highest incidence in lungs exposed to quartz DQ12, to a lesser degree in those treated with quartz DQ12 + PVNO and carbon black. The macrophages appeared to be generally intact in the lungs instilled with coal dust and amorphous SiO_2_. In the amorphous SiO_2_—exposed lungs -instead of particle-laden—large foamy multivesicular macrophages were detected in scattered alveoli. These small vesicles are known to be derived from phagocytosed surfactant material, respectively fragmented lamellar bodies of the alveolar type II cells (Mahlke, 2002; Dungworth et al., 1992b).

The interstitial changes were marked by mixed inflammatory cell infiltrations and interstitial fibrosis frequently associated with bronchiolo-alveolar epithelial hyperplasia in all experimental groups but with considerable differences in severity and incidence as demonstrated in Figure 2.

Bronchiolo-alveolar hyperplasia was seen in all experimental groups. A spectrum of severity grades ranging from very subtle and focal epithelial hyperplasia to severe or preneoplastic lesions some with transitions to neoplasia were evaluated. Explicit differences in severity and incidence were observed related to the treatment group (Figure 2). The highest incidence and severity grades were found in the quartz-exposed lungs, the difference was signifcant (tested with the Mann-Whitney *U*-Test) compared to the other experimental groups. The lowest mean severity grade was seen in the control and coal dust-treated group with small and single foci whereas carbon black-and amorphous SiO_2_ -treated animals showed more frequent and bigger sized bronchiolo-alveolar hyperplasias.

The most distinct inflammatory cell infiltration, interstitial fibrosis and diffuse/multifocal alveolar lipoproteinosis were seen in the quartz-treated group (Figure 2).

The addition of PVNO reduced the incidence and severity of these lesions significantly. In the lungs of carbon black exposed rats these findings were statistically significant less severe than those of the quartz-treated rats (Figure 2).

Those animals treated with coal dust and amorphous SiO_2_ showed no alveolar lipoproteinosis, only occasionally very small scattered foci were seen in single rats. Minimal inflammation and little interstitial fibrosis were observed in coal dust exposed lungs. The mean severity of Inflammation and interstitial fibrosis in amorphous SiO_2_ exposed lungs was comparable to those of the quartz DQ12 + PVNO and carbon black-treated groups after 9 months (published elsewhere by Ernst et al., 2005) but regressed clearly after 30 months as seen in Figure 2.

In this carcinogenicity study cholesterol granulomas were observed in 96% of the quartz-treated rats, 95% of the quartz + PVNO-treated, 81% of the carbon black-treated and in 21% of the amorphous SiO_2_ and in 6% of the coal dust-treated rats.

Squamous cell metaplasia—often associated with distinct pulmonary inflammatory reactions including increased stromal collagen—became evident after 24 months of exposure and was exclusively observed in the lungs of quartz-, quartz + PVNO- and carbon black-treated rats. Just a single animal of the coal dust-treated animals showed focal slight squamous metaplasia (Figure 2).

Goblet-cell metaplasia also seen as response to chronic irritation respectively chronic inflammation showed a similar distribution; they were mainly formed in the quartz-, quartz + PVNO- and carbon black-exposed lungs. In the coal dust- and amorphous SiO_2_ - treated groups this finding was minimally developed and detected in single animals.

## Discussion

As expected, the exposure of rats to crystalline silica (quartz DQ12) elicits the greatest magnitude and progression of pulmonary inflammatory reactions, fibrosis and neoplastic response. These findings are consistent with those of previous studies and can be used as appropriate positive control reference material, which is essential for the evaluation of instillation studies (Warheit et al., 2007).

The addition of PVNO clearly reduced the incidence and severity of quartz DQ12-induced pulmonary inflammatory reactions and fibrosis. In respect of neoplastic and preneoplastic lung lesions the incidence of lung tumors as well as the incidence of preneoplasias in rats treated with quartz DQ12 and PVNO was almost 50% lower compared to rats treated with quartz DQ 12 only. These findings indicate a preventive effect of subcutaneously injected PVNO on the development of cancerous lung lesions after exposure to quartz DQ12 in rats by partly inhibiting the development DQ 12 related lung toxicity.

Under the experimental conditions of this study (30 times intratracheal instillation of synthetic amorphous SiO_2_ at intervals of 14 days with subsequent 8 or 9 months of recovery) the carcinogenic potential of ultra-fine amorphous SiO_2_ (Aerosil 150®) was observed at a statistically significant degree (*p* = 0.0257) compared to the control group. This effect was confirmed by using the method of multiple-step sectioning (Kolling et al., 2008). These tumors occurred late in life and the diameter of three tumors out of 5 was less than 2 mm. For the assessment of the so far unknown neoplastic response of the rat lung to ultrafine amorphous silica the toxicity of this material in the lung and the differences in the dose rate and distribution of particulate material delivered by frequent instillation versus inhalation into the lung have to be taken into account (Oberdörster et al., 2005). The purpose of the repeated instillations of amorphous SiO_2_ in the present study was to maintain a chronic particle effect over 60 weeks in spite of the rapid clearance of these ultrafine particles. But, it has to be taken into account that the toxicity of amorphous SiO_2_ in the lung is much stronger than the toxicity known from poorly soluble particles.

Regarding the toxicologically significant changes in non-cancer endpoints, the exposure to ultrafine amorphous SiO_2_ induced a pronounced transient pulmonary inflammation with formation of interstitial fibrotic granulomas presumably a sequence of acute alveolitis following epithelial damage at the sites of particle deposition (Ernst et al., 2002; Ernst et al., 2005). Compared to the more persistent and progressive pulmonary inflammation of carbon black, the granulomatous inflammation of amorphous silica resolved during the observation period by leaving only focal fibrotic scar tissue due to the rapid clearance rate of amorphous SiO_2_ (Ernst et al., 2002).

Notably is the absence of squamous metaplasia in the group exposed to amorphous SiO_2_. Squamous metaplasia is considered as the most obvious form of cellular response to injury and is commonly associated with severe chronic inflammatory reactions.

The reason that no increased mutation frequency was observed in epithelial lung cells after 13 weeks of exposure to 50 mg/m^3^ amorphous silica although a strong inflammatory response was reported (Johnston et al., 2000) could possibly be explained by the strong cytotoxic effect of amorphous silica leading to increased cell death also of mutated cells. For the tumor induction relevant mutations will probably occur later in life time of the exposed animals as in our study when the amorphous silica has been cleared out of the lung and tissue regeneration and scar developing processes have been started. The small size of the lung tumors found in our rats exposed to amorphous silica after 29 months experimental time indicates that these tumors may have started to develop rather late in life time of these animals.

In addition, the causation of the tumors observed in rats treated with amorphous silica should be handled with care as it can not be excluded that the high frequency of intratracheal instillations may have added to the development of neoplasias (Driscoll et al. 2000). Although, Pott et al. (1987; 1994) showed in control groups of previous experiments conducted with Wistar rats that 20 intratracheal instillations of physiological saline did not induce lung tumors.

In the control group and in the experimental group exposed to coal dust no primary lung tumors were detected neither after the routine examination of single sections nor after the supplemental evaluation of step sections through the entire lung (Kolling et al., 2008). In summary, the low concentration of fine lean coal dust (10 × 1 mg) applied by repeated instillation failed to induce lung tumors. Exposure to higher concentrations (6 × 11 mg) of the same fine lean coal dust induced a tumor incidence of 57% (Pott and Roller, 2005; Mohr et al., 2006). The results of this study seem to fit in a non-linear dose-response relationship and indicate the existence of a threshold below which no tumor inducing effect might occur in the rat lung.

**Figure 7:**
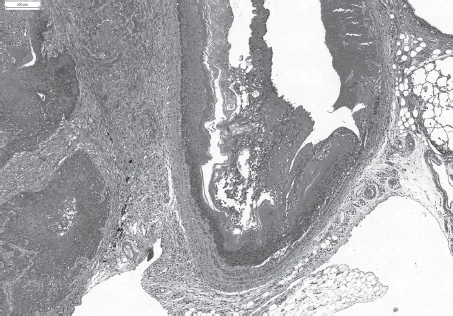
Invading squamous cell carcinoma after repeated intratracheal instillation of ultrafine carbon black (PRINTEX® 90). H&E × 10. (See colour version of this figure online at www.informahealthcare.com/iht)

**Figure 8:**
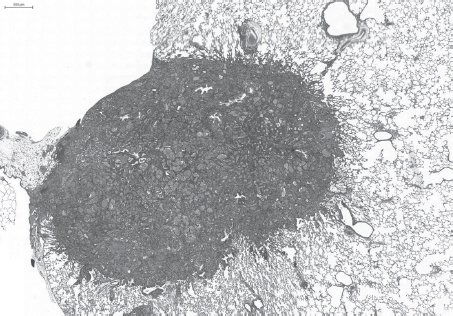
Bronchiolo-alveolar carcinoma with metaplasia to goblet cells and mucin production after repeated intratracheal instillation of ultrafine amorphous SiO_2_ (Aerosil). H&E. (See colour version of this figure online at www.informahealthcare.com/iht)

Comparing all treatment groups the highest tumor incidence was associated with the most distinct inflammation, fibrosis and epithelial hyperplasia as observed in the quartz DQ12 exposed rats. Those animals treated with carbon black (Figure 7), amorphous SiO_2_ (Figure 8) and coal dust showed statistically significantly less severe lesions in all non-cancer endpoints and accordingly a lower tumor frequency or even no tumors as seen in the coal dust treated group showing only minimal inflammation and fibrosis (Figures 3–6). Although rats exposed to coal dust and those to amorphous SiO_2_ showed similar extent of inflammation only the amorphous SiO_2_ treated animals developed lung tumors. The reason might be the strong initial acute pulmonary inflammatory response to ultrafine amorphous SiO_2_, which can conceivably trigger long-term effects, despite a low biopersistence of the particles. This presumption is supported by the significant increase in interstitial fibrosis and bronchiolo-alveolar hyperplasias of the amorphous SiO_2_ treated rats compared to the coal dust treated rats (Figure 2). A further evidence for the higher acute toxicity of amorphous SiO_2_ was the results from bronchioalveolar lavage fluid (BALF) 9 months after first instillation. They showed that coal dust induced the lowest concentrations of BAL leukocytes (200,000) and PMN [65,000 (40-fold higher than the control group)] of all treatment groups whereas the highest cell concentrations of 560,000 leukocytes (192 fold higher than the control) and 310,000 PMN were determined in the amorphous SiO_2_ group during the repeated instillation period (Ernst et al., 2002).

Our data show -when using a qualitative scoring system for specific non-cancerous endpoints of lung toxicity such as inflammation, fibrosis, epithelial hyperplasia and squamous metaplasia- a relationship between tumor frequencies and increasing scores of these changes.

These findings refer to a mechanism of secondary genotoxicity [pathway of genetic damage resulting from the oxidative DNA attack by reactive oxygen/nitrogen species, generated during particle-elicited inflammation (Schins and Knaapen, 2007)] such as inflammation-induced carcinogenesis (Oberdörster et al., 1996; Oberdörster, 1997).

Moreover, current available literature data indicate that the tumorigenesis of poorly soluble particles involves a mechanism of secondary genotoxicity at doses that induce inflammation *in vivo* (Greim et al., 2001).

The aspect of secondary genotoxicity originates from observations that various poorly soluble particles are carcinogenic in rat lungs, rather irrespective of their chemical composition, but obviously after chronic high exposures that are associated with overload and persistent inflammation (Greim et al., 2001; Borm et al., 2004). Of major importance for hazard assessment, secondary genotoxicity is considered to involve a threshold: its value is set to be determined by the exposure concentration that will trigger inflammation and overwhelm anti-oxidant and DNA damage repair capacities in the lung (Greim et al., 2001). Lung tumors were never found in rats when pulmonary inflammation was absent during chronic inhalation of particles (Oberdörster et al., 1997). There is a direct relationship between chronic inflammation and carcinogenesis in exposed rats (Oberdörster et al., 1997). In the OECD guidelines for carcinogenicity studies is noted that some non-genotoxic carcinogens induce tumors as a secondary event following a toxicological event that has a threshold. These substances do not present a carcinogenic hazard at doses that do not produce the primary toxicological event. Using the example of carbon black Driscoll et al. (1996) had demonstrated that a dose-dependent increased mutation frequency of alveolar epithelial cells was found that paralleled the responses of inflammatory cell influx. These results are consistent with the existence of a threshold (Oberdörster et al., 1996). Collectively, these findings suggest that particle induced pulmonary carcinogenicity in rats is mediated via an indirect mechanism involving chronic inflammation, generation of reactive oxygen species and subsequent oxidative effects on DNA in target cells.

One of the major conclusions of the current study is that a low dose of fine coal dust resulted in minimal pulmonary inflammation and failed to induce lung tumors. This finding indicates the existence of a threshold below which no carcinogenic effect might occur.
